# Implementation and user acceptance of a domestic robot-assisted surgical training system: a pilot educational study at the first clinical medical college of Jinan university

**DOI:** 10.3389/fmed.2026.1767561

**Published:** 2026-09-15

**Authors:** Wenming Shao, Jianfan Chen, Yidan Wang, Weijie Liu, Jixin Yang, Hui Zhao, Mingya Zhang

**Affiliations:** 1 Department of Emergency Medicine, The First Affiliated Hospital of Jinan University, Guangzhou, China; 2 Department of Urology, the First Affiliated Hospital, Jinan University, Guangzhou, China; 3 Clinical Skills Comprehensive Training Center, The First Affiliated Hospital of Jinan University, Guangzhou, China; 4 School of Mechanical Engineering and Automation, Dalian Polytechnic University, Dalian, China; 5 Department of Hepatobiliary Surgery, Guangzhou Eighth People’s Hospital, Guangzhou Medical University, Guangzhou, China

**Keywords:** free surgery, post grad medical education, robot, robot-Assisted surgical systems, simulation medical education

## Abstract

**Objective:**

Robot-assisted surgical systems are being increasingly utilized in the field of surgery, and have been reported to offer enhanced 3D visualization, force feedback, superior ergonomics for the surgeon, and improved operative precision, which collectively may contribute to minimally invasive outcomes and expedited postoperative recovery. However, the integration of robot-assisted technology into surgical training curricula in Chinese medical colleges remains limited. This pilot implementation study aims to evaluate the usability, acceptance, and perceived educational value of the Jingfeng Multi-port Robot-Assisted Surgical System in a structured surgical training program in China.

**Method:**

The validity of the robot-assisted surgical system was first assessed using the System Usability Scale (SUS). Participants then received practical training in applying the robotic technology to abdominal surgical procedures. Subsequently, a questionnaire was administered to collect their perceptions and evaluations regarding the integration of this technology into surgical practice.

**Result:**

The mean SUS score was 80.58 ± 12.04. Questionnaire responses indicated positive participant perceptions regarding system usability and potential educational value, reflecting user acceptance rather than objectively measured educational effectiveness. Participants reported favorable views on perceived enhancements in learning efficiency, system characteristics, and ease of operation. Notably, male users gave significantly higher ratings (5.94 ± 1.26) than their female counterparts (4.78 ± 1.36) on the “Ease of operation” factor (*P* = 0.011), suggesting a stronger perception of the system's user-friendliness among male participants.

**Conclusion:**

In this observational pilot study, participants reported positive perceptions of the training mode, including perceived potential benefits for learning efficiency, motivation, and enjoyment. These perceptions do not establish causal effects of robotic training on educational outcomes.

## Introduction

With the rapid advancement of technology, robot-assisted surgical systems have become an increasingly important component of modern medicine (1). In China, robot-assisted surgical systems are being adopted more widely in the healthcare sector, particularly in surgery, where robot-assisted systems are now regarded as important complementary tools (2). Despite initial steps to adopt robot-assisted systems as training platforms in Chinese medical schools, their widespread integration remains limited. As of 2023, only 15 elite institutions had incorporated this technology into surgical training programs, accounting for just 5.8% of all medical schools in the country, despite a noticeable trend toward gradual adoption (3). The First Clinical Medical College of Jinan University is one such institution that has integrated robot-assisted surgical systems into its curriculum. These training platforms provide valuable opportunities for surgical trainees to gain hands-on experience while also serving as a platform for young surgeons to engage with internationally advanced medical technology. Through these systems, trainees can perform repetitive practice in a virtual environment, allowing them to master surgical procedures and enhance their technical skills, and facilitate the transfer of these skills into real clinical practice (4). While robot-assisted surgical systems have been extensively validated in clinical practice across various surgical specialties (5–11), their integration into surgical training curricula remains less established.

Given the established clinical utility of robotic systems, their application in surgical teaching and training is both necessary and innovative (12). While traditional surgical training primarily relies on animal models and cadaver dissection (13)—methods that are effective yet constrained by limited resources, high costs, and an inability to fully replicate live surgical conditions (14)—robot-assisted systems offer an alternative by providing a highly simulated virtual environment. This allows students to engage in repetitive practice under safe and controlled conditions (15). Additionally, robotics can provide students with personalized learning paths through data analysis and feedback mechanisms (15, 16), helping learners acquire surgical skills more efficiently (17). This innovative teaching model has the potential to enhance educational efficiency and may provide support for preparing future surgeons. Nevertheless, although there is a strong rationale for incorporating robotic systems into medical curricula, their reliability and educational effectiveness still require further validation. International research on robot-assisted surgical education has mainly focused on simulation-based training, transfer of skills to actual surgical procedures, and comparisons with conventional training methods (14, 18). Randomized controlled trials have shown that virtual reality-based robotic simulation significantly improves residents’ instrument proficiency, spatial awareness, and procedural compliance (19). Furthermore, structured, competency-based curricula coupled with objective metrics (e.g., operation time, error rates, motion economy) are essential for effective training (20). However, existing literature predominantly focuses on imported systems such as the da Vinci platform in Western settings (21), which may not align with medical training realities in China. Moreover, the high costs of acquisition, maintenance, and clinical use have restricted access to imported systems (22). In recent years, domestically developed surgical robots have emerged (2), offering comparable outcomes at reduced cost (23) and creating a need for educational validation of these domestic platforms. Despite these advances, simulation-based training in robot-assisted surgery, as a novel teaching approach, has not yet been widely adopted in China as a routine preclinical training method for surgeons. It is important to note that this study is primarily educational and implementation-oriented, focusing on the feasibility, usability, and perceived value of integrating a domestic robotic system into a training curriculum, rather than on clinical outcomes. Therefore, in this pilot study, we utilized the China Jingfeng Robot-Assisted Surgical System as the training platform. Based on this system, we developed a surgical training protocol and trained surgeons at or above the attending level at the First Affiliated Hospital of Jinan University in Guangzhou, China. Subsequently, we collected feedback from these trainees through a questionnaire survey to evaluate their perceptions and initial acceptance of the Jingfeng robotic surgical system.

## Materials and methods

### Robot settings

The robotic platform used in this study was the Jingfeng Robot-Assisted Surgical System (Jingfeng Medical Technology Co., Ltd., Shenzhen, China). This robotic system consisted of three major components: a surgeon's console, a patient-side robotic arm system, and an imaging system (Figure 1). During operation, the lead surgeon sat at the control console and manipulated the robotic arms using hand controllers and foot pedals to perform precise and complex surgical maneuvers. Meanwhile, the assistant operated the imaging system at the patient side to support the procedure. The VR surgical training pod was equipped with a high-precision head-mounted display featuring medical-grade 4 K resolution dual OLED screens, force feedback controllers with six-degree-of-freedom electromagnetic induction handles, dual infrared cameras for optical tracking, a master control workstation, a physiological signal synchronization system, and a foot switch integrated into the multimodal interaction terminal. The software platform used was Jingfeng VR-SurgSim, which included modules for intelligent three-dimensional reconstruction, force-feedback simulation, training and assessment, a multi-scenario training library, and data management and collaboration.

**Figure 1:**
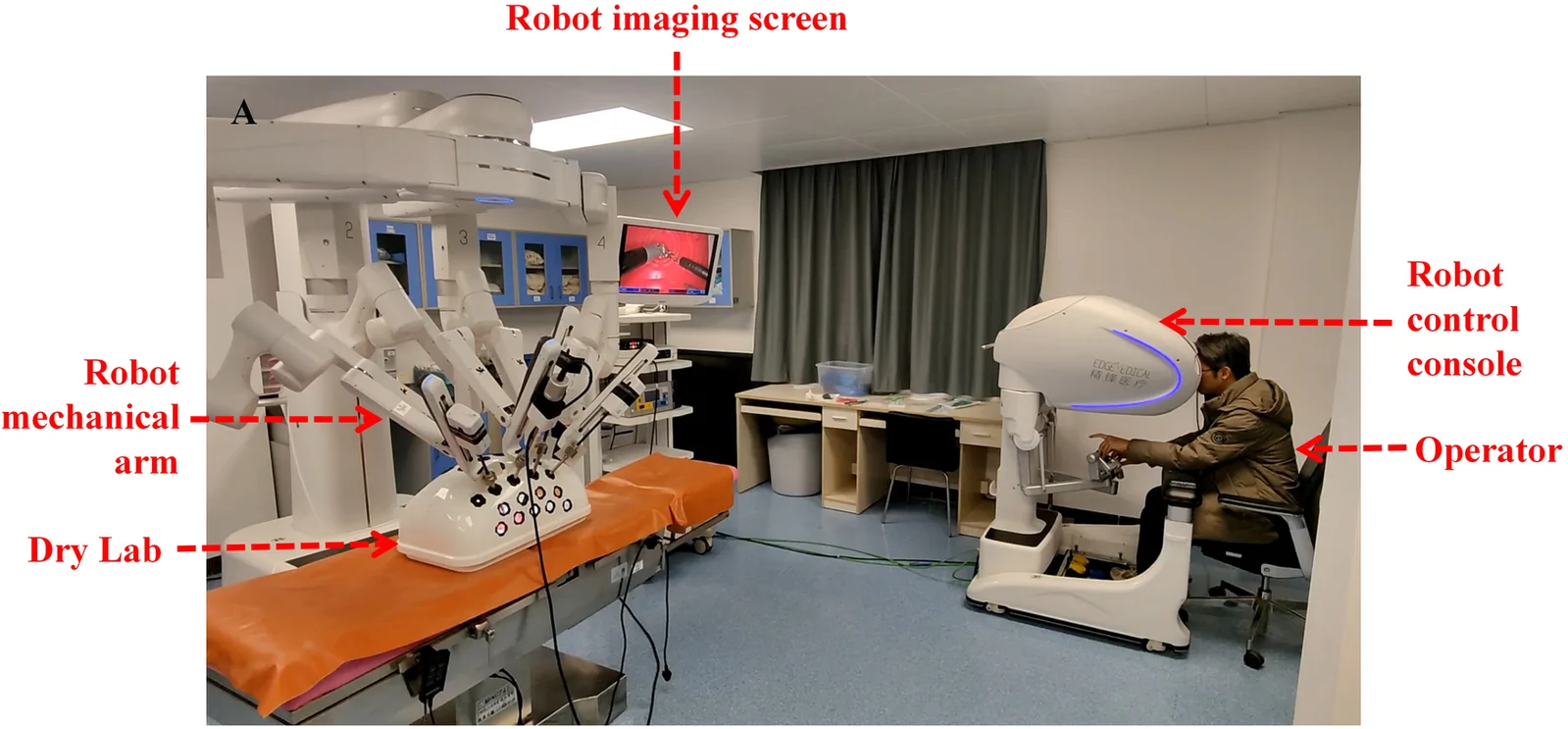
Robot setup used for learning laparoscopic surgery during student practices. **(A)** The Robot system consists of a control console, mechanical arm, robot imaging screen.

### The application of robot-assisted technology systems in laparoscopic training

This study used the “Robotic-Assisted Laparoscopic Surgery” training module to explore the potential applications of robot-assisted surgical systems in laparoscopic surgical education. To ensure effective training, a dual-learner collaborative model was adopted. Each trainee pair, consisting of a primary operator and an assistant, completed a 240-minute hands-on training session. The basic training program included the following structured tasks: (1) a bead-threading task designed to develop hand-eye coordination and fine motor control; (2) an S-shaped metal ring wire-guiding task intended to improve spatial path planning and instrument navigation skills; (3) skin simulator exercises, including incision, suturing, and knot tying, to practice fundamental surgical maneuvers; and (4) live animal model surgery, in which participants performed one or two complete procedures on a porcine model to integrate these skills in a realistic operative setting. A total of 36 clinicians participated voluntarily without any incentives. The training objectives were to help participants master fundamental surgical procedures, become familiar with operational protocols for the robot-assisted surgical system, and implement standardized workflows for robot-assisted laparoscopic surgery. Steps A-F in Figure 2 illustrate the fundamental operational procedures of the robotic surgical system. The detailed workflow is as follows: 1. System Preparation: The assistant and technician complete the assembly of the robotic surgical system and connect the required instruments (patient-side robotic arm, imaging cart, etc.) (Figure 1). 2. Console Operations: The primary surgeon performs the following tasks at the console: Task 1: String colored beads of different shapes onto a cord to make a bracelet. The target requirement is to complete 12 beads within 60 s (Figure 2A**)**. Task 2: Guide the flexible steel wire through the array of S-shaped metal rings within 3 min (Figures 2B,C). Task 3: Perform basic surgical procedures (incision, suturing, knot tying) on the skin simulator (Figures 2D–F). Task 4: complete one or two surgical procedures on a live porcine model (Figure 3). These four progressive tasks were incorporated into a structured 2-day robotic surgery training curriculum designed to provide comprehensive coverage and repeated practice of core robotic surgical skills. The first day focused on deconstructed basic skill training, encompassing system familiarization, camera navigation, precision targeting, suturing and knot tying, and the use of energy devices. The second day emphasized comprehensive procedural application, during which trainees performed complete surgical procedures, such as intestinal anastomosis and partial gastrectomy, on live porcine models. This curriculum was designed to systematically reinforce essential technical competencies, including camera manipulation, instrument handling, suturing, knot tying, and the use of stapling and sealing devices. Before the training session, all participants received a 15-minute system orientation and watched a standardized instructional video. All practical procedures were closely supervised by teaching assistants and recorded in full.

**Figure 2:**
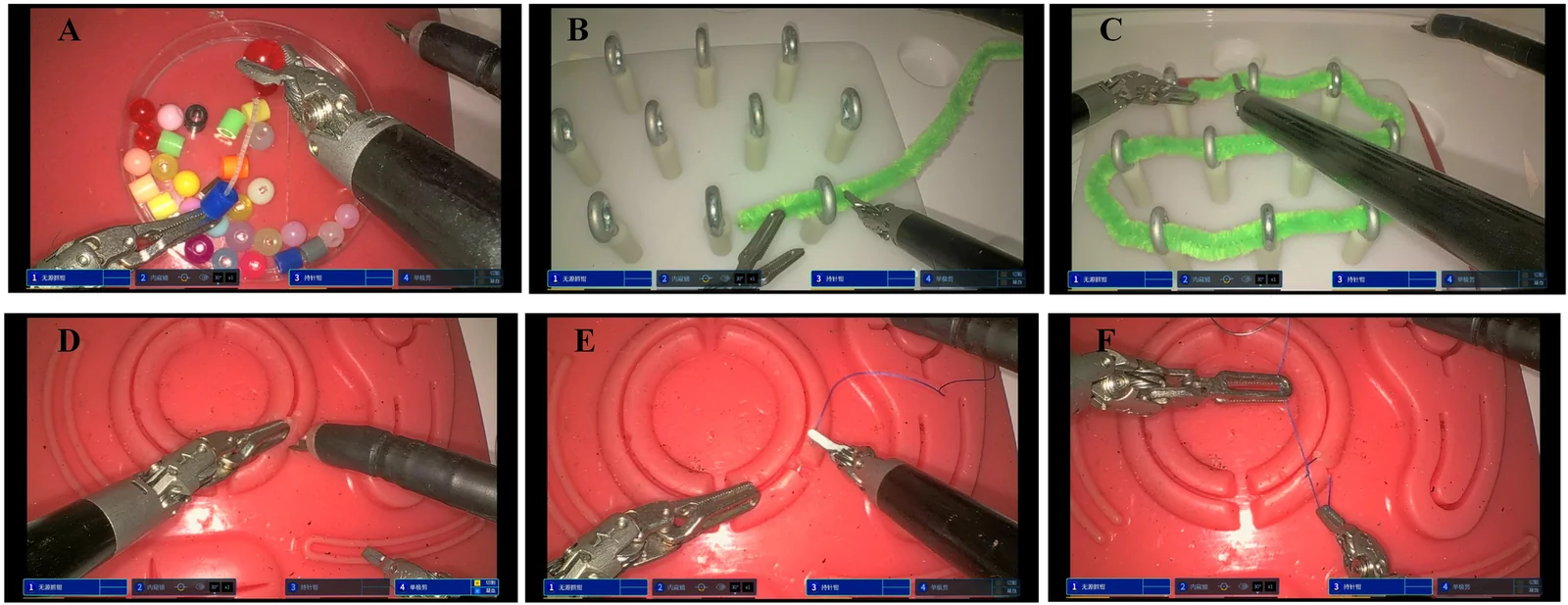
Basic operation training steps for robotic surgical procedures. **(A)**Thread different-shaped and colored beads onto a string to make a bracelet; **(B,C)** Guide the flexible steel wire through the "S"-shaped metal ring array; **(D,E,F)** Performing basic surgical procedures (incision, suturing, knot-tying) on the skin simulator; Carry out robotic surgical operations on live pig models.

**Figure 3:**
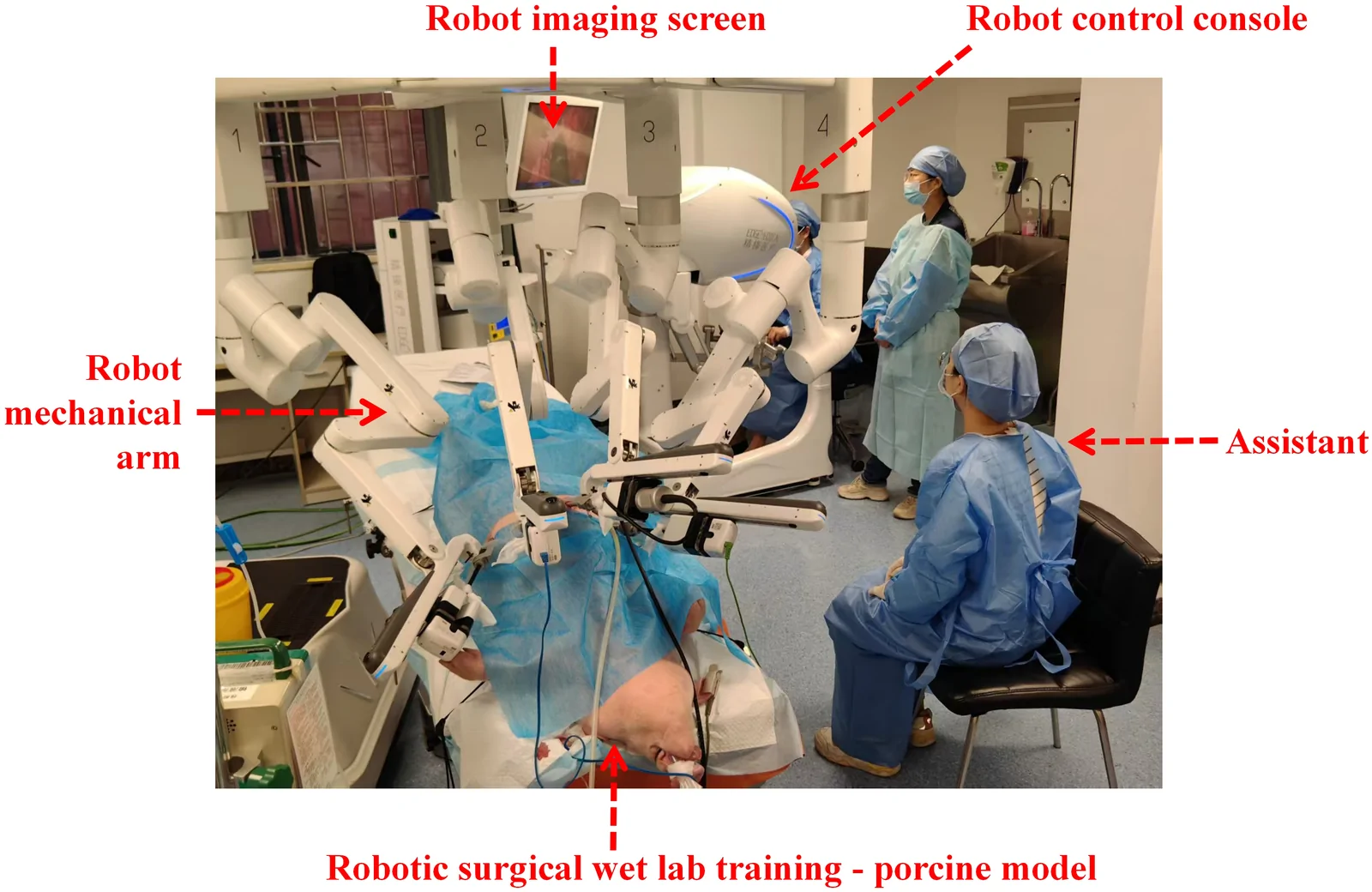
A surgical student (operator) is using the robot system.

### Participants and questionnaire

#### Inclusion and exclusion criteria

Participants were included if they met the following criteria: (1) held the rank of attending physician or above in a surgical department at the First Affiliated Hospital of Jinan University; (2) were actively engaged in clinical surgical practice; (3) possessed adequate visual acuity (corrected or uncorrected) and hand dexterity to operate the robotic console; and (4) provided written informed consent and agreed to complete the full training protocol. Participants were excluded if they: (1) reported a history of severe motion sickness, vertigo, or vestibular disorders that would preclude the use of a head-mounted VR display; (2) had experienced a hand or wrist injury or surgical procedure within the preceding 3 months that could impair fine motor control; or (3) were unable to commit to the full 240-minute hands-on training session.

Consistent with the educational and implementation-oriented nature of this study, the trainees participating in the robot-assisted surgical system training were attending physicians or above from the surgical departments of the First Affiliated Hospital of Jinan University. Participants were recruited voluntarily through an announcement issued by the hospital’ s Medical Affairs Office. A total of 38 surgeons initially enrolled in the robot-assisted surgery training program. Two participants withdrew before completing the full training, resulting in 36 participants who completed both the training and the questionnaire (completion rate: 94.7%). All subsequent analyses were based on these 36 participants. This recruitment strategy was chosen because, as a pilot implementation study, we sought to obtain expert usability and educational value assessments from experienced surgeons before introducing the system to junior trainees. Consequently, the findings reflect the perspectives of experienced clinicians and may not be generalizable to residents, fellows, or medical students. A structured questionnaire was designed to collect feedback on their training experience and the perceived usability and acceptance of the robot-assisted surgical systems. It is important to note that these instruments measure subjective perceptions and user satisfaction, not objective educational outcomes or competency-based skill acquisition. Information on participants’ gender and prior experience with robotic systems was collected. Upon completion of the training, each participant was asked to complete the questionnaire through SoJump, a widely used online survey platform in China. The studies involving human participants were reviewed andapproved by The First Affiliated Hospital of Jinan University. Written informed consent was obtained fromthe individuals to participate in this study and for the publication ofany potentially identifiable images or data included in this article. Anesthesia and euthanasia: Before the surgical training procedure, the pigs were anesthetized with an intramuscular combination of ketamine (5 mg/kg), medetomidine (0.06 mg/kg), and butorphanol (0.2 mg/kg). Following induction of anesthesia, the animals were endotracheally intubated, and general anesthesia was maintained with 2% isoflurane. The animals were monitored throughout the surgical training procedure by trained personnel. At the completion of the training procedure, the animals were maintained under deep isoflurane anesthesia and euthanized by intravenous administration of pentobarbital (30 mg/kg). Cessation of heartbeat and respiration, as well as bilateral fixed dilated pupils, were observed to confirm death prior to disposal. All animal procedures were performed in accordance with the protocol approved by the institutional animal care and use committee. (approval number KY-2026-042). Two questionnaires were employed in the survey. The first questionnaire was the SUS, which was used to assess participants’ experiences with and acceptance of the robot-assisted system and equipment. The SUS is a reliable and widely used instrument for assessing the usability of various products and services (24, 25). It comprises 10 items, each rated on a 5-point Likert scale ranging from “Strongly Disagree” to “Strongly Agree”. The overall SUS score is calculated by summing the scores for all items and then multiplying the total by 2.5, thereby converting the original total score to a scale ranging from 0 to 100. The second questionnaire was a custom-designed instrument to gather participants’ feedback on the application of robotic-assisted system technology in surgical education, including perceived ease of use, immersion in the virtual environment, support for learning, and perceived educational potential. The 17 items were initially generated based on a systematic review of the robotic surgical education literature and mapped to four core domains: (1) perceived ease of use and system operability; (2) immersion and sensory fidelity in the virtual environment; (3) perceived educational value and learning efficiency; and (4) intention to adopt and integrate robotic training into surgical education. Content validity was established through an expert panel review by three independent experts (two senior attending surgeons with >10 years of robotic/laparoscopic experience and one medical education specialist), who rated the relevance of an initial 21-item pool on a 4-point scale (1 = not relevant, 2 = somewhat relevant, 3 = quite relevant, 4 = highly relevant). Four items with an item-level Content Validity Index (I-CVI) < 0.78 were revised or removed, yielding the final 17-item questionnaire with a scale-level CVI (S-CVI/Ave) of 0.94. Face validity and clarity were subsequently confirmed through pilot testing with eight attending surgeons not participating in the main study; two senior instructors conducted structured cognitive interviews to identify ambiguous terminology, double-barreled questions, and culturally inappropriate phrasing. Based on this feedback, five items were reworded and two items were split to avoid double-barreled constructs. The final questionnaire comprised 17 items. Participants rated these items on a 5-point Likert scale ranging from 1 (“strongly disagree”) to 5 (“strongly agree”). To enhance accessibility for Chinese participants, the questionnaire was developed in Mandarin Chinese, and English translations are provided in the Supporting Information appendix (Supplementary Appendix S1). Participation in the survey was voluntary.

#### Data analysis

Validity and reliability testing, descriptive statistics, and correlation analyses were performed using SPSS version 22.0. Cronbach's *α* was employed to assess internal consistency of questionnaire responses. Given the small sample size, exploratory factor analysis was conducted solely as a preliminary, hypothesis-generating exercise to tentatively explore potential latent dimensions underlying participants' feedback. Mann–Whitney U tests were used to compare questionnaire item scores between groups stratified by gender and prior experience. No adjustment for multiple comparisons (e.g., Bonferroni or false discovery rate) was applied given the exploratory nature of this pilot study; consequently, the reported *p*-values for gender comparisons should be interpreted with caution and as hypothesis-generating only. Effect sizes were calculated using Cohen's d, defined as the difference between two group means divided by the pooled standard deviation: Cohen's d = (*M2 - M1*)/SD_pooled_, where SDpooled = $\sqrt{(\mathrm{SD}1^{2}+\mathrm{SD}2^{2}}$)/2. Effect sizes were interpreted using Cohen's (1988) conventions: small (d ≈ 0.2), medium (d ≈ 0.5), and large (d ≈ 0.8). Power analysis was performed using G*Power software (Franz Faul, Germany). In this study, *α* was set at 0.05 and beta (*β*) at 0.20, which is four times the *α* level, considering the relative severity of committing a *β* error. Enter sample sizes for different groups to calculate power (1 -*β*). The desired power is set at 0.80. Continuous data are presented as mean ± standard deviation (SD), with *P* < 0.05 considered statistically significant.

## Results

### Participant characteristics

A total of 36 attending physicians or senior surgeons from seven surgical specialties completed the study. The cohort was evenly balanced by gender (50% male, 50% female), with a mean age of 42.17 years and an average of 14.6 years of surgical practice. Prior experience with robotic systems was reported by 27.78% of participants, predominantly involving observation or assistant roles rather than primary console operation. Detailed baseline characteristics are presented in Table 1.

**Table 1:** Baseline characteristics of the study participants (*n* = 36). Data are presented as *n* (%) or mean ± standard deviation (SD).

Characteristics	*n* (%) or Mean ± SD
Male	18 (50.0)
Female	18 (50.0)
Age (years)	42.17 ± 2.92
Years of surgical practice	14.6 ± 4.8
Surgical specialty	
General surgery	9 (25.0)
Urology	7 (19.4)
Hepatobiliary surgery	6 (16.7)
Orthopedic surgery	5 (13.9)
Neurosurgery	4 (11.1)
Gynecology	3 (8.3)
Cardiothoracic surgery	2 (5.6)
Prior robotic experience	
Yes	10 (27.78)
No	26 (72.22)
Type of prior experience (*n* = 10)	
Observation only	6 (60.0)
Bedside assistant	3 (30.0)
Simulation training	1 (10.0)
Primary console surgeon	0 (0)

Data are presented as mean ± standard deviation, or number (percent).

#### Evaluation of robot-assisted surgical system implementation

According to the established SUS benchmark, a score above 68 is considered above average (26), while a score exceeding 80.3 places a system within the top 10% of usability ratings (26). In this study, the mean SUS score was 80.58 ± 12.04 (Table 2).

**Table 2:** The participants’ evaluation of jingFeng’ s surgical robots application using system usability scale (SUS).

Questions	Mean	SD	Skewness
1. I think that as long as the courses I take have robotic equipment, I will choose to use robots.	3.83	1.31	−1.23
2. Robots have complex and unnecessary functions.	1.80	0.92	0.71
3. Robots are easy to use.	3.80	1.06	−0.86
4. Requires technician's help to use the machine.	3.2	1.03	−0.83
5. The individual functions of the robot are well integrated.	4.00	0.74	−0.54
6. There are too many places where the robots contradict each other.	1.73	0.69	0.41
7. It takes a very short time to learn to use a robot.	3.33	1.15	−0.72
8. Robots are a pain in the ass to use.	2.10	0.96	0.29
9. I felt confident in the robot.	3.93	0.78	−0.34
10. A lot of learning is necessary to be proficient in operating this system.	4.50	0.86	−2.60

Data are presented as Mean and Standard Deviation (SD). The skewness statistic describes the asymmetry of the data distribution. A *p*-value of < 0.05 was considered statistically significant for all analyses.

#### Analysis of gender differences in the Use of robot-assisted systems for surgical procedures

To evaluate participants’ subjective perceptions of using robotic-assisted surgical systems for surgeon training, all trainees were invited to complete a post-training questionnaire regarding their experience with the robotic system (Supporting Information,  Supplementary Appendix S1). Of the 38 enrolled participants, 36 completed the full training and returned valid questionnaires (response rate: 94.7%). The questionnaire showed good internal consistency, with a Cronbach's *α* of 0.893. The survey collected information on participants’ demographic characteristics, hands-on experience with the robotic surgical system, evaluations of system performance, and perspectives on its integration into surgical education. The final sample included 18 male and 18 female participants. The mean age was 42.78 ± 3.66 years for male participants and 41.56 ± 2.06 years for female participants. Participants with prior experience accounted for 27.78% of the total sample. Among all questionnaire items, the lowest mean score was recorded for Question 2, which assessed whether participants could learn to use the robotic system independently without guidance from a teaching assistant, with a mean score of 2.39 ± 0.96 (Table 2). Additionally, we compared responses to questionnaire items based on participants' gender and prior experience with robotic-assisted surgical systems. Results indicated four items were associated with gender (Table 4), specifically: “Robot operation is easy to master”[Question 1(Q1); uncorrected *P* = 0.011], “Manipulation to accurately find the anatomical location of a patient's lesion”（Question 4[Q4]; *P* = 0.023），“In regard to learning outcome, I think that Robot system is better than the traditional operation in laboratory”[Question 1(Q10); *P* = 0.021]，“The robotic experience has helped my proficiency in surgical operations”（Question 1[Q12]; *P* = 0.027). Male participants scored higher than female participants (Q1: 4.21 ± 0.81 vs. 3.44 ± 0.92, Cohen's d = 0.89, large effect; Q4: 3.78 ± 0.94 vs. 3.06 ± 0.87, Cohen's d = 0.79, large effect; Q10: 3.94 ± 1.11 vs. 3.17 ± 0.79, Cohen's d = 0.80, large effect; Q12: 4.06 ± 1.00 vs. 3.39 ± 0.70, Cohen's d = 0.78, large effect). Regarding these issues, whether they are related to experienced individuals, the results indicate no statistically significant association (Table 3). However, given the small sample size (*n* = 18 per group) and the absence of multiple comparison adjustment, these findings should be considered exploratory and may reflect Type I error.

**Table 3:** Evaluations of jingFeng's surgical robots from participants of different genders and robotic experiences.

Gender							Experience			
Questions	Total	male	female	*Z*	*P*	Cohen's d	No	Yes	*Z*	*P*
*N*	36 (100%)	18 (50%)	18 (50%)				26 (27.8%)	10 (72.2%)		
1. Robot operation is easy to master.	3.83 ± 0.94	4.21 ± 0.81	3.44 ± 0.92	2.69	0.011*	0.89	3.85 ± 0.97	3.80 ± 0.92	0.13	0.896
2. Experimental tutorial on learning to use a robot alone without the guidance of a teaching assistant.	2.39 ± 0.96	2.11 ± 1.08	2.67 ± 0.77	−1.78	0.085	—	2.46 ± 0.99	2.20 ± 0.92	0.75	0.464
3. Experienced real sensations such as touch and vibration in a virtual environment.	3.58 ± 1.27	3.5 ± 1.54	3.67 ± 0.97	−0.39	0.701	—	3.54 ± 1.27	3.70 ± 1.34	−0.33	0.747
4. Manipulation to accurately find the anatomical location of a patient's lesion.	3.42 ± 0.97	3.78 ± 0.94	3.06 ± 0.87	2.39	0.023*	0.79	3.35 ± 0.98	3.60 ± 0.97	−0.70	0.491
5. I just repeated the process.	3.86 ± 1.05	3.89 ± 1.02	3.83 ± 1.10	0.16	0.876	—	3.81 ± 1.13	4.00 ± 0.82	−0.56	0.578
6. Thirty minutes of robotics is enough time for me to get the hang of it.	3.36 ± 0.99	3.22 ± 1.17	3.50 ± 0.79	−0.84	0.54	—	3.54 ± 0.81	2.90 ± 1.29	1.46	0.170
7.Using the robotics system made me more motivated to learn the section.	4.25 ± 0.60	4.33 ± 0.77	4.17 ± 0.38	0.82	0.417	—	4.19 ± 0.63	4.40 ± 0.52	−1.01	0.324
8. Robotic systems make the learning process fun.	4.25 ± 0.73	4.33 ± 0.77	4.17 ± 0.71	0.68	0.502	—	4.19 ± 0.80	4.40 ± 0.52	−0.92	0.368
9. Robotic system clearly recognizes the fine structure of human organs.	4.17 ± 0.81	4.00 ± 1.03	4.33 ± 0.49	−1.24	0.226	—	4.15 ± 0.88	4.20 ± 0.63	−0.17	0.863
10. In regard to learning outcome, I think that Robot system is better than the traditional operation in laboratory	3.56 ± 1.03	3.94 ± 1.11	3.17 ± 0.79	2.43	0.021*	0.80	3.54 ± 1.07	3.60 ± 0.97	−0.17	0.870
11. Learning is more effective with a robotic system.	4.14 ± 0.83	3.94 ± 1.06	4.33 ± 0.49	−1.42	0.168	—	4.19 ± 0.90	4.00 ± 0.67	0.70	0.491
12. The robotic experience has helped my proficiency in surgical operations.	3.72 ± 0.91	4.06 ± 1.00	3.39 ± 0.70	2.32	0.027*	0.78	3.65 ± 0.94	3.90 ± 0.88	−0.74	0.469
13. I think robotic systems should be combined with traditional experiments.	4.39 ± 0.55	4.28 ± 0.57	4.50 ± 0.51	−1.22	0.230	—	4.35 ± 0.56	4.50 ± 0.53	−0.77	0.452
14. Recognizing that robotic systems are ideally suited as surgical aids.	4.36 ± 0.59	4.39 ± 0.70	4.33 ± 0.49	0.28	0.84	—	4.35 ± 0.63	4.40 ± 0.52	−0.26	0.795
15.The robot system looks technologically sophisticated and spatially realistic.	3.50 ± 1.08	3.56 ± 0.92	3.44 ± 1.25	0.30	0.763	—	3.46 ± 1.10	3.60 ± 1.07	−0.34	0.735
16. I would spend more time learning with robots.	4.44 ± 0.61	4.39 ± 0.70	4.50 ± 0.51	−0.54	0.590	—	4.38 ± 0.64	4.60 ± 0.52	−1.05	0.307
17. Would you like other science programs to adopt robotics.	3.33 ± 1.17	3.44 ± 1.20	3.22 ± 1.17	0.56	0.577	—	3.35 ± 1.20	3.30 ± 1.16	0.11	0.917

Data are presented as mean ± standard deviation. Inter-group comparisons were performed using the Mann–Whitney U test. A *p*-value of < 0.05 was considered statistically significant, as indicated by *.

**Table 4:** Summary of the exploratory factor analysis of the questionnaire on robots application on surgical practice.

Questions	Factor 1	Factor 2	Factor 3
12. The robotic experience has helped my proficiency in surgical operations.	0.872		
9. Robotic system clearly recognizes the fine structure of human organs.	0.871		
10. Robotic systems make learning more efficient.	0.838		
11. Learning is more effective with a robotic system.	0.828		
8. Robotic systems make the learning process fun.	0.778		
7. Using the robotics system made me more motivated to learn the section.	0.752		
13. I believe that robotic systems should be combined with traditional experiments.	0.706		
14. Recognizing that robotic systems are ideally suited as surgical aids.	0.697		
4. Manipulation to accurately find the anatomical location of a patient's lesion.		0.813	
1. Robot operation is easy to master.		0.75	
15. The robot system looks technologically sophisticated and spatially realistic.		0.703	
5. I just tested this workflow again.		0.68	
17. Would you like other science programs to adopt robotics.		0.614	
16. I would spend more time learning with robots.		0.533	
3. Experienced real sensations such as touch and vibration in a virtual environment.		0.49	
2. Experimental tutorial on learning to use a robot			0.833
6. Thirty minutes of robotics is enough time for me to get the hang of it.			0.718
r (reliability)	0.938	0.867	0.712
% of Variance	36.668	22.924	10.342
Key words	Enhancing learning efficiency	System superiority	Easy to operate system

The table displays the factor loadings of each questionnaire item onto the three tentatively extracted factors. Factor loadings represent the correlation between the items and the factors. The internal consistency reliability (Cronbach's *α*) for each factor, the percentage of variance explained, and the conceptually derived keywords for each factor are also provided. It must be emphasized that with only 36 participants for 17 items (approximately 2.1 participants per item), the sample size falls well below conventional recommendations (5–10 participants per item) for reliable factor extraction. Therefore, this three-factor solution is exploratory and potentially unstable, and should not be interpreted as a validated psychometric structure. A *p*-value of < 0.05 was considered statistically significant for all analyses in this study.

#### Principal component analysis (PCA)

An exploratory factor analysis was conducted on the 36 training questionnaires completed by participants. Given that the sample size (*n* = 36) for 17 items yields approximately 2.1 participants per item — substantially below the conventional recommendation of 5–10 participants per item for reliable factor extraction — the results should be interpreted as preliminary and hypothesis-generating only. The analysis tentatively suggested a three-factor structure (Table 4), with the following dimensions emerging: participants' perception that robotic application enhances learning efficiency (Factor 1) (r = 0.938, variance explained = 36.668%), the technical superiority of the robot-assisted system (Factor 2) (r = 0.867, variance explained = 22.924%), and the ease of use of robot operation (Factor 3) (r = 0.712, variance explained = 10.342%). These three factors collectively account for 69.934% of the total variance. However, the stability of this three-factor solution is uncertain given the inadequate sample size, and the model fit should not be considered satisfactory in a psychometric validation sense. Based on these exploratory dimensions, we further explored gender differences across the derived factors. Through t-tests conducted on the three tentative factors, the “Easy to operate system” factor showed uncorrected statistically significant differences between genders, with *P* < 0.05 between the two groups (male 5.94 ± 1.26, female 4.78 ± 1.36,Cohen's d = 0.88, large effect) (Table 5). Given the small sample size (*n* = 18 per group) and the exploratory nature of this comparison, this observation should be interpreted with caution and requires replication in a larger cohort.

**Table 5:** Descriptive statistics of the three tentatively identified factors, classified by gender and experience (t-test).

Questions(*n*, %)	Enhancing learning efficiency	System superiority	Easy to operate system
Total (36, 100%)	30.3 ± 4.2	28.5 ± 4.1	5.36 ± 1.42
**Gender**			
Male (18, 50%)	31.2 ± 4.5	28.9 ± 5.5	5.94 ± 1.26
Female (18,50%)	29.5 ± 3.8	28.1 ± 2.1	4.78 ± 1.36
*t*	1.20	0.60	−1.59
*P*	0.237	0.553	0.011*
Cohen's d	—	—	0.88
**Experience**			
Yes (10, 72.2%)	31.0 ± 3.6	29.0 ± 2.6	5.40 ± 1.51
No (26, 27.8%)	30.1 ± 4.4	28.3 ± 4.6	5.35 ± 1.41
*t*	−0.65	−0.60	1.35
*P*	0.524	0.552	0.923

Data are presented as mean ± standard deviation. Inter-group comparisons were performed using independent samples t-tests, with the assumption of homogeneity of variances verified by Levene's test. These comparisons are based on exploratory factors derived from a sample size (*n* = 36) inadequate for reliable factor extraction, and should therefore be interpreted with caution. A *p*-value of < 0.05 was considered statistically significant, as indicated by *.

## Discussion

Robot-assisted surgical systems have been extensively validated in clinical practice across a range of surgical fields. Compared with conventional laparoscopy, these systems may facilitate more precise intraoperative maneuvers, reduce neurovascular injury, and enable smaller incisions, which may in turn support improved postoperative recovery (27). Consequently, robot-assisted procedures have been associated with lower rates of intraoperative complications, such as bleeding and organ injury (28), as well as reduced short-term and long-term postoperative adverse outcomes (27). However, previous studies have also suggested that performing robotic surgery without adequate simulation-based training may lead to insufficient surgical skills, teamwork issues, and operational errors, thereby potentially compromising patient safety. Such deficiencies may manifest as increased risks of complications, prolonged surgical duration, or diminished overall therapeutic efficacy (14, 29). Therefore, simulation-based training is considered an important component in the safe implementation of robot-assisted surgical systems in clinical practice (14). As an educational implementation study, our primary aim was to assess user acceptance and perceived educational value rather than to measure objective skill improvement.

In this study, participants reported a high level of acceptance of the robotic system in the training setting. Overall, participants generally perceived the system as offering potential benefits for learning motivation, training efficiency, and operational skill development, and broadly supported its integration with conventional teaching approaches. Regarding gender, most questionnaire items showed no significant differences by gender or prior experience, suggesting that the system may be broadly acceptable among users from diverse backgrounds. At the same time, participants gave relatively low ratings to the item concerning independent learning without the guidance of a teaching assistant. This finding suggests that participants perceived a need for enhanced tutorial design or guidance mechanisms, as well as structured self-directed tutorials, to lower the barriers to unsupervised learning.

It is critical to emphasize that all findings reported herein are derived entirely from subjective questionnaire responses. These findings include perceived benefits for learning efficiency, motivation, and operational skill development. In the absence of objective skill-based assessments (e.g., operative time, error rates, motion economy metrics) and a comparator group receiving conventional laparoscopic or simulation-based training, no inference can be drawn regarding the superior educational efficacy of the robotic curriculum. These results should therefore be interpreted as indicators of user acceptance, perceived usability, and subjective educational value, rather than as evidence of objectively superior training outcomes.

The findings indicate that participants perceived potential benefits of robotic systems for surgical training efficiency and effectiveness, as well as perceived enhancements in learner motivation and engagement. In this sense, the system may serve as a valuable adjunct to traditional experimental teaching methods rather than a replacement for them. Participants also acknowledged the technological features of robotic systems, particularly their reported utility in precise anatomical localization, consistent with previous research findings (30). Participants found the system's operation relatively easy to master, providing a good sense of spatial awareness and a certain degree of realistic tactile feedback. Participants reported that these perceived features encouraged them to invest more time in learning the system, which may be associated with greater willingness to consider its broader adoption in surgical education. Therefore, this outcome suggests the study participants' recognition of its technical performance and perceived educational value, which may provide a basis for further exploration of its promotion and application in the surgical field. However, these subjective perceptions of acceptance should not be interpreted as evidence of objective educational effectiveness, which would require competency-based metrics such as OSATS, GEARS, task completion time, error rates, and motion economy analysis.

An uncorrected gender difference was observed in the perceived ease of system operation, with male participants reporting higher scores (Table 5). Given the very small sample size (18 males versus 18 females), the absence of adjustment for multiple comparisons, and the lack of mechanistic data, we do not interpret this finding as evidence of a true gender effect. Rather, we offer the following untested hypotheses for future investigation: first, that ergonomic features of the console and controllers (e.g., grip size, button placement, force feedback intensity) may differentially accommodate male hand anthropometry; second, that prior exposure to video gaming or spatial navigation tasks may influence initial comfort with the interface; and third, that differences in the initial learning curve may exist. These speculations are not supported by empirical data from the present study and should not be regarded as established explanations. If replicated in larger, adequately powered samples, such observations might highlight opportunities for more gender-inclusive simulator design, such as adjustable hardware components, customizable force settings, or enhanced tutorial guidance. Future research incorporating biomechanical assessments, usability testing across genders, and longitudinal training outcomes is warranted to test these hypotheses. It should also be emphasized that the absence of significant gender differences in the factors of “improved learning efficiency” and “system superiority” (*P* > 0.05) suggests that any ease-of-operation difference may be minor and of uncertain practical significance. This suggests that user evaluations of these aspects were relatively consistent and not significantly differentiated by gender factors in this sample. Based on usage experience, prior experience had no significant impact on evaluations of all three factors (*P* > 0.05). It is important to acknowledge that all participants in this pilot study were attending physicians or senior surgeons with substantial clinical experience. While their expert feedback is valuable for initial system validation and curriculum design, their learning curves, usability perceptions, and educational needs likely differ substantially from those of residents, fellows, or undergraduate medical students. Therefore, the positive perceptions reported herein cannot be assumed to apply to less experienced learners. Future studies should evaluate the system's usability and educational impact across different training levels, particularly among surgical residents and medical students who represent the primary target population for robotic surgical training in the broader context of medical education. This indicates that both novice and experienced users share largely consistent perceptions regarding the system's learning efficiency enhancement, system performance, and ease of use. This finding suggests the system was perceived as having strong accessibility and consistent user experience, which could support its consideration for a broad range of applications.

The application of robot-assisted surgical technology systems in training surgical practice skills may represent a promising development in surgical education, with potential to contribute to standardized workflow (14). However, the present findings reflect user acceptance rather than validated educational effectiveness. While the present training protocol was implemented with attending physicians, its modular design—ranging from basic manipulation to procedural simulation—could be adapted for residents and fellows through paced skill progression and enhanced tutorial support. According to this process, surgical residents or interns should undergo objective training by senior and experienced attending surgeons or higher-level surgeons who have mastered robotic-assisted surgical techniques in their respective fields at the hospital. Only after passing this training may they perform surgical procedures on patients under supervision. Providing high-quality educational services to meet student needs presents a global challenge for surgical training centers. Robot-assisted surgical technology systems may offer a useful tool to help address this challenge. robotic simulation may provide a repeatable and high-fidelity environment for foundational skills acquisition before trainees perform procedures in real operating rooms (14). This suggests that early-stage technical skill acquisition might potentially be supported by robotic simulation, which could reduce exclusive reliance on operating-room exposure. Furthermore, robotic simulation may potentially reduce the time experienced surgeons must dedicate to teaching foundational skills. However, it must be emphasized that many other fundamental competencies of a qualified surgeon, such as precise anatomical knowledge, intraoperative decision-making ability, adaptability to unforeseen circumstances, and other skills, are equally critical, not merely the practical skills of operating a robotic system. To effectively cultivate surgical talent through robotic systems, merely providing robotic hardware is far from sufficient. Specialized simulation software tailored to different surgical specialties (such as minimally invasive surgery and neurosurgery), realistic training modules, and corresponding professional guidance are equally indispensable. Participants perceived that robot-assisted surgery may offer potential value to surgical education, but it is recognized that it may not fully replace high-quality traditional surgical teaching methods such as gross anatomy, observational learning, and progressive hands-on practice under direct mentor guidance (31–33). Consequently, determining how to best integrate robotic and traditional surgical training hinges on a clear understanding of their respective merits and drawbacks. Defining this complementary relationship is crucial for developing a hybrid model that capitalizes on the strengths of both, ultimately paving the way for a more efficient pathway to producing comprehensively competent surgeons.

Robot-assisted surgical training may serve as a potentially effective tool for skill acquisition and a contributing component of the ever-expanding digital health ecosystem. By combining high-fidelity simulation with data capture, such systems provide a technology-enabled educational model that is less constrained by time and location than traditional training approaches. This aligns with the broader digital health paradigm, which aims to enhance healthcare system resilience during public health crises (34). For instance, these platforms possess remote supervision potential, enabling expert physicians to provide virtual guidance to trainees. This ensures the continuity of high-quality education, unimpeded by geographical constraints or disparities in institutional resources. Furthermore, the inherent capacity of these systems to record operational metrics—such as movement efficiency and error rates—lays the foundation for data-driven skill development. It facilitates both personalized feedback delivery and objective proficiency tracking, marking a significant advancement compared to conventional subjective assessment methodologies. The developmental trajectory of robotic surgical training parallels the well-documented adoption curve of telemedicine. Just as telemedicine has revolutionized clinical care (35) through digital enablement, improving healthcare accessibility, continuity, and system efficiency, robot-assisted simulation strongly supports surgical education by establishing scalable, standardized, and resilient training models. Both paradigms entail a shift away from purely physical, location-bound interactions toward digitally enhanced, reproducible, and analytically rich processes. This synergistic effect highlights the potential value of integrating robotic systems into modern medical curricula: beyond serving as a tool for skill training, they may contribute as one element toward constructing a future-oriented, data-driven, and equitable surgical education ecosystem.

## Limitations

It is important to acknowledge several limitations. Firstly, no control group was included, and no objective skill-based assessment was performed. The outcome measures relied exclusively on self-reported satisfaction and perceptions (SUS and a custom perception questionnaire), without objective competency-based metrics such as task completion time, error rates, motion economy, OSATS scores, GEARS assessment, or transfer of skills to clinical performance. Consequently, the study demonstrates user acceptance rather than educational effectiveness, and the study cannot determine whether the robotic curriculum provides superior educational outcomes compared with conventional laparoscopic or simulation-based training, nor can it establish causal relationships between robotic training and objective skill improvement. As a result, the study was unable to directly compare practical skill outcomes between robot-assisted training and conventional training approaches. This gap underscores the need for additional evidence to robustly confirm the technology's educational impact. In future studies, we plan to incorporate a randomized controlled trial design. This will involve establishing a control group that undergoes traditional training and employing objective skill assessment metrics, such as operation time, error rates, and global rating scales. Secondly, the restricted sample size (*n* = 36), single-center design, and exclusive inclusion of attending physicians or senior surgeons substantially limit the generalizability of our findings. Although the results demonstrate perceived potential for robotic assistance in surgical training, they cannot be extrapolated to residents, fellows, or undergraduate medical students, nor to other institutions with different resource levels or curricula. The title has been revised to accurately reflect that this is a pilot implementation study focused on experienced clinicians rather than typical undergraduate medical learners. Future research will therefore enroll a substantially expanded and more diverse cohort across various institutions, regions, and training levels (including residents and medical students), to strengthen the robustness and generalizability of the findings. Thirdly, this training protocol has not yet been formally evaluated in resident or fellows populations. Future work should involve developing a tiered curriculum with more finely graduated progression from basic skills to core procedural modules, tailored to the distinct needs of trainees at different levels. Fourthly, future iterations of this training protocol must incorporate objective competency-based assessment tools, including OSATS, GEARS, task completion time, error rates, and motion economy metrics, to determine whether this robotic training system produces measurable educational effectiveness beyond subjective user acceptance. Fifthly, the exploratory factor analysis conducted on the 17-item questionnaire with only 36 participants (approximately 2.1 participants per item) falls substantially below the conventional threshold of 5–10 participants per item required for reliable factor extraction. Consequently, the reported three-factor solution is unstable and should be considered preliminary and hypothesis-generating rather than a validated psychometric structure. The factor loadings, variance explained, and the derived factor-based gender comparisons should all be interpreted with considerable caution. Future research must validate the questionnaire's factor structure in a substantially larger and independent sample before these dimensions can be used with confidence in subsequent studies.

## Conclusion

In this observational pilot study, participants reported positive perceptions of the training mode, including perceived potential benefits for learning efficiency, motivation, and enjoyment. These perceptions do not establish causal effects of robotic training on educational outcomes.

## Data Availability

The original contributions presented in the study are included in the article/Supplementary Material, further inquiries can be directed to the corresponding authors.

## References

[1] Lanfranco AR Castellanos AE Desai JP Meyers WC. Robotic surgery: a current perspective. Ann Surg. (2004) 239(1):14–21. 10.1097/01.sla.0000103020.19595.7d 14685095 PMC1356187

[2] Gu L Yin C Jia T He K Ma X Zhang X. Robotic surgery in China. Innovation (Camb). (2023) 4(5):100499. 10.1016/j.xinn.2023.100499 37705606 PMC10495633

[3] Xu D Lou W Li M Xiao J Wu H Chen J. Current Status of robot-assisted surgery in the clinical application of trauma orthopedics in China: a systematic review. Health Sci Rep. (2022) 5(6):e930. 10.1002/hsr2.930 36381405 PMC9642816

[4] Raison N Harrison P Abe T Aydin A Ahmed K Dasgupta P. Procedural virtual reality simulation training for robotic surgery: a randomised controlled trial. Surg Endosc. (2021) 35(12):6897–902. 10.1007/s00464-020-08197-w 33398587 PMC8599326

[5] Wandvik C Greil ME Colby S Gautam D Mazur MD. Limitations of current robot-assisted pedicle screw insertion systems. Neurosurg Focus. (2024) 57(6):E14. 10.3171/2024.9.Focus24545 39616634

[6] MacLean L Hersh AM Bhimreddy M Jiang K Davidar AD Weber-Levine C. Comparison of accuracy, revision, and perioperative outcomes in robot-assisted spine surgeries. Systematic Review and Meta-Analysis. J Neurosurg Spine. (2024) 41(4):519–31. 10.3171/2024.4.Spine23917 38968628

[7] Molliqaj G Paun L Nouri A Girod PP Schaller K Tessitore E. Role of robotics in improving surgical outcome in spinal pathologies. World Neurosurg. (2020) 140:664–73. 10.1016/j.wneu.2020.05.132 32445895

[8] Gautam D Vivekanandan S Mazur MD. Robotic spine surgery: systematic review of common error types and best practices. Oper Neurosurg. (2025) 28(3):295–302. 10.1227/ons.0000000000001293 39037253

[9] Khalsa SSS Mummaneni PV Chou D Park P. Present and future spinal robotic and enabling technologies. Oper Neurosurg. (2021) 21(Suppl 1):S48–56. 10.1093/ons/opaa338 34128072

[10] Jourdes F Valentin B Allard J Duriez C Seeliger B. Visual haptic feedback for training of robotic suturing. Front Robot AI. (2022) 9:800232. 10.3389/frobt.2022.800232 35187094 PMC8849007

[11] Pham MH Brown NJ. Evolution of robotic spine surgery technologies. Neurosurgery. (2025) 96(3s):S75–83. 10.1227/neu.0000000000003351 39950787

[12] Kim Y Genevriere E Harker P Choe J Balicki M Regenhardt RW. Telerobotic neurovascular interventions with magnetic manipulation. Sci Robot. (2022) 7(65):eabg9907. 10.1126/scirobotics.abg9907 35417201 PMC9254892

[13] Sinha A West A Vasdev N Sooriakumaran P Rane A Dasgupta P. Current practises and the future of robotic surgical training. Surgeon. (2023) 21(5):314–22. 10.1016/j.surge.2023.02.006 36932015

[14] Walshaw J Fadel MG Boal M Yiasemidou M Elhadi M Pecchini F. Essential components and validation of multi-specialty robotic surgical training curricula: a systematic review. Int J Surg. (2025) 111(4):2791–809. 10.1097/js9.0000000000002284 39903561 PMC12175810

[15] Radi I Tellez JC Alterio RE Scott DJ Sankaranarayanan G Nagaraj MB. Feasibility, effectiveness and transferability of a novel mastery-based virtual reality robotic training platform for general surgery residents. Surg Endosc. (2022) 36(10):7279–87. 10.1007/s00464-022-09106-z 35194662 PMC8863393

[16] Ma R Lee RS Nguyen JH Cowan A Haque TF You J. Tailored feedback based on clinically relevant performance metrics expedites the acquisition of robotic suturing skills-an unblinded pilot randomized controlled trial. J Urol. (2022) 208(2):414–24. 10.1097/ju.0000000000002691 35394359

[17] Howard KK Makki H Novotny NM Mi M Nguyen N. Value of robotic surgery simulation for training surgical residents and attendings: a systematic review protocol. BMJ Open. (2022) 12(6):e059439. 10.1136/bmjopen-2021-059439 35701063 PMC9198707

[18] Wu CC Gong B Zhang X Yang J. Global research landscape of robot-assisted surgical training: a 35-year bibliometric and visualization analysis. Int J Surg. (2026) 112:10359–73. 10.1097/js9.0000000000004716 41524090 PMC13105644

[19] Li T Yan J Gao X Liu H Li J Shang Y. Using virtual reality to enhance surgical skills and engagement in orthopedic education: systematic review and meta-analysis. J Med Internet Res. (2025) 27:e70266. 10.2196/70266 40446337 PMC12143859

[20] Basile G Gallioli A Diana P Gallagher A Larcher A Graefen M. Current standards for training in robot-assisted surgery and endourology: a systematic review. Eur Urol. (2024) 86(2):130–45. 10.1016/j.eururo.2024.04.008 38644144

[21] Kim HJ Elises JCR Castillo CJ Aljameel N Alzahrani F Sulaiman O. Robot-Assisted retrograde intrarenal surgery: a Mini review of the literature on robotic surgery platforms for endoscopic stone surgery. Eur Urol Focus. (2025) 11(5):716–20. 10.1016/j.euf.2025.06.011 40617785

[22] Verma A Hadaya J Richardson S Vadlakonda A Ramezani R Revels S’S. The presence of a cost-volume relationship in robotic-assisted thoracoscopic lung resections. Ann Surg. (2023) 278(2):e377–e81. 10.1097/sla.0000000000005699 36073775

[23] Liu Y Wang Y Wang C Wang X Zhang X Yang Y. Comparison of short-term outcomes of robotic-assisted radical colon cancer surgery using the kangduo surgical robotic system and the Da vinci si robotic system: a prospective cohort study. Int J Surg. (2024) 110(3):1511–8. 10.1097/js9.0000000000000976 38085814 PMC10942201

[24] Bangor A Kortum P Miller J. Determining what individual sus scores mean: adding an adjective rating scale. J Usability Stud. (2009) 4(3):114–23.

[25] Brooke J. Sus-a quick and dirty usability scale. Usability Evaluation in Industry. (1996) 189(194):4–7.

[26] Sauro J Lewis JR. Quantifying the User Experience: Practical Statistics for User Research. Cambridge, MA: Morgan Kaufmann (2016).

[27] Zaman S Mohamedahmed AYY Yassin NA. Minimally invasive surgery for inflammatory bowel disease: a systematic review and meta-analysis of robotic versus laparoscopic surgical techniques. J Crohns Colitis. (2024) 18(9):1522–3. 10.1093/ecco-jcc/jjae065 38780033

[28] Müller PC Breuer E Nickel F Zani S Kauffmann E De Franco L. Robotic distal pancreatectomy: a novel standard of care? Benchmark values for surgical outcomes from 16 international expert centers. Ann Surg. (2023) 278(2):253–9. 10.1097/sla.0000000000005601 35861061

[29] Campbell KK Abreu AA Zeh HJ Daniel WC Palter VN Bishop SJ. Using or black box technology to determine quality improvement outcomes for *in-situ* timeout and debrief simulation. Ann Surg. (2026) 283(1):122–9. 10.1097/sla.0000000000006438 38989569

[30] Liu C Liu Y Xie R Li Z Bai S Zhao Y. The evolution of robotics: research and application progress of dental implant robotic systems. Int J Oral Sci. (2024) 16(1):28. 10.1038/s41368-024-00296-x 38584185 PMC10999443

[31] Woldehana NA Jung A Parker BC Coker AM Haut ER Adrales GL. Clinical outcomes of laparoscopic vs robotic-assisted cholecystectomy in acute care surgery. JAMA Surg. (2025) 160(7):755–62. 10.1001/jamasurg.2025.1291 40397430 PMC12096326

[32] Ellison EC Stain SC Lillemoe KD. Introduction to the contemporary assessment and recommendations to enhance surgical education and training: reports of the subcommittees of the blue ribbon committee ii. Ann Surg. (2025) 281(1):1–2. 10.1097/sla.0000000000006495 39109426

[33] Boal MWE Anastasiou D Tesfai F Ghamrawi W Mazomenos E Curtis N. Evaluation of objective tools and artificial intelligence in robotic surgery technical skills assessment: a systematic review. Br J Surg. (2024) 111(1). 10.1093/bjs/znad331 37951600 PMC10771126

[34] Tahmasbi F Toni E Javanmard Z Kheradbin N Nasiri S Sadoughi F. An overview of reviews on digital health interventions during COVID- 19 era: insights and lessons for future pandemics. Arch Public Health. (2025) 83(1):129. 10.1186/s13690-025-01590-8 40346715 PMC12063330

[35] Asadi H Toni E Ayatollahi H. Application of telemedicine technology for cardiovascular diseases management during the COVID-19 pandemic: a scoping review. Front Cardiovasc Med. (2024) 11:1397566. 10.3389/fcvm.2024.1397566 39188320 PMC11345180

