# Supplementary material for: Implementation and user acceptance of a domestic robot-assisted surgical training system: a pilot educational study at the first clinical medical college of Jinan university
Source: Front Med (Lausanne). 2026 Sep 15;13:1767561. doi: 10.3389/fmed.2026.1767561 (PMC13619467; doi:10.3389/fmed.2026.1767561)
Supplement: Supplementary file 1 [file Supplementaryfile1.docx]

**Survey Questionnaire on Evaluations of Jingfeng Robotic Equipment by Trainee Surgeons at the JNU First Affiliated Hospital**

Dear participating surgeons: The confidentiality of your responses is guaranteed. They will be used for no other purpose than this research. In accordance with ethical standards, all data collected will be anonymized, ensuring that no individual participant is identifiable.

Thank you very much for participating in our robotics equipment experience. This survey will be used to improve our robotics equipment and enhance teaching quality. Please rate the robot you just experienced using the following scale: (1: Strongly Disagree, 2: Disagree, 3: Neutral, 4: Agree, 5: Strongly Agree). The survey consists of 10 questions. Thank you again for your participation.

**Gender: male /female**

**Age:**

1.I think that as long as the courses I take have robotic equipment, I will choose to use robots.

1

2

3

4

5

2.Robots have complex and unnecessary functions.

1

2

3

4

5

3.Robots are easy to use.

1

2

3

4

5

4.Requires technician’s help to use the machine.

1

2

3

4

5

5.The individual functions of the robot are well integrated.

1

2

3

4

5

6.There are too many places where the robots contradict each other.

1

2

3

4

5

7.It takes a very short time to learn to use a robot.

1

2

3

4

5

8.Robots are a pain in the ass to use.

1

2

3

4

5

9.I felt confident in the robot.

1

2

3

4

5

10.A lot of learning is necessary to be proficient in operating this system.

1

2

3

4

5

**Survey Questionnaire on the Integration of Robot-Assisted System Technology and Surgical Education**

Please rate the following questions based on your experience with the robot learning process using the scale: (1: Strongly Disagree, 2: Disagree, 3: Neutral, 4: Agree, 5: Strongly Agree)

**Gender: male /female**

**Age:**

**Experience (referred to those who had prior hands-on experience with robotic-assisted surgical systems): yes/no**

1.Robot operation is easy to master.

1

2

3

4

5

2.Experimental tutorial on learning to use a robot alone without the guidance of a teaching assistant.

1

2

3

4

5

3.Experienced real sensations such as touch and vibration in a virtual environment.

1

2

3

4

5

4.Manipulation to accurately find the anatomical location of a patient's lesion.

1

2

3

4

5

5.I just repeated the process.

1

2

3

4

5

6.Thirty minutes of robotics is enough time for me to get the hang of it.

1

2

3

4

5

7.Using the robotics system made me more motivated to learn the section.

1

2

3

4

5

8.Robotic systems make the learning process fun.

1

2

3

4

5

9.Robotic system clearly recognizes the fine structure of human organs.

1

2

3

4

5

10.In regard to learning outcome, I think that Robot system is better than the traditional operation in laboratory

1

2

3

4

5

11.Learning is more effective with a robotic system.

1

2

3

4

5

12.The robotic experience has helped my proficiency in surgical operations.

1

2

3

4

5

13.I think robotic systems should be combined with traditional experiments.

1

2

3

4

5

14.Recognizing that robotic systems are ideally suited as surgical aids.

1

2

3

4

5

15.The robot system looks technologically sophisticated and spatially realistic.

1

2

3

4

5

16.I would spend more time learning with robots.

1

2

3

4

5

17.Would you like other science programs to adopt robotics.

1

2

3

4

5
